# Supplementary material for: The CDK4/6 Inhibitor Palbociclib Inhibits Estrogen-Positive and Triple Negative Breast Cancer Bone Metastasis In Vivo
Source: Cancers (Basel). 2023 Apr 8;15(8):2211. doi: 10.3390/cancers15082211 (PMC10137281; doi:10.3390/cancers15082211)
Supplement: Supplementary file 1 [file cancers-15-02211-s001.zip › cancers-2272186-supplementary.pdf]

Supplementary Materials

The CDK4/6 Inhibitor Palbociclib Inhibits Estrogen-Positive and Triple Negative Breast Cancer Bone Metastasis In Vivo

Lubaid Saleh, Penelope D. Ottewell, Janet E. Brown, Steve L. Wood, Nichola J. Brown, Caroline Wilson, Catherine Park, Simak Ali and Ingunn Holen

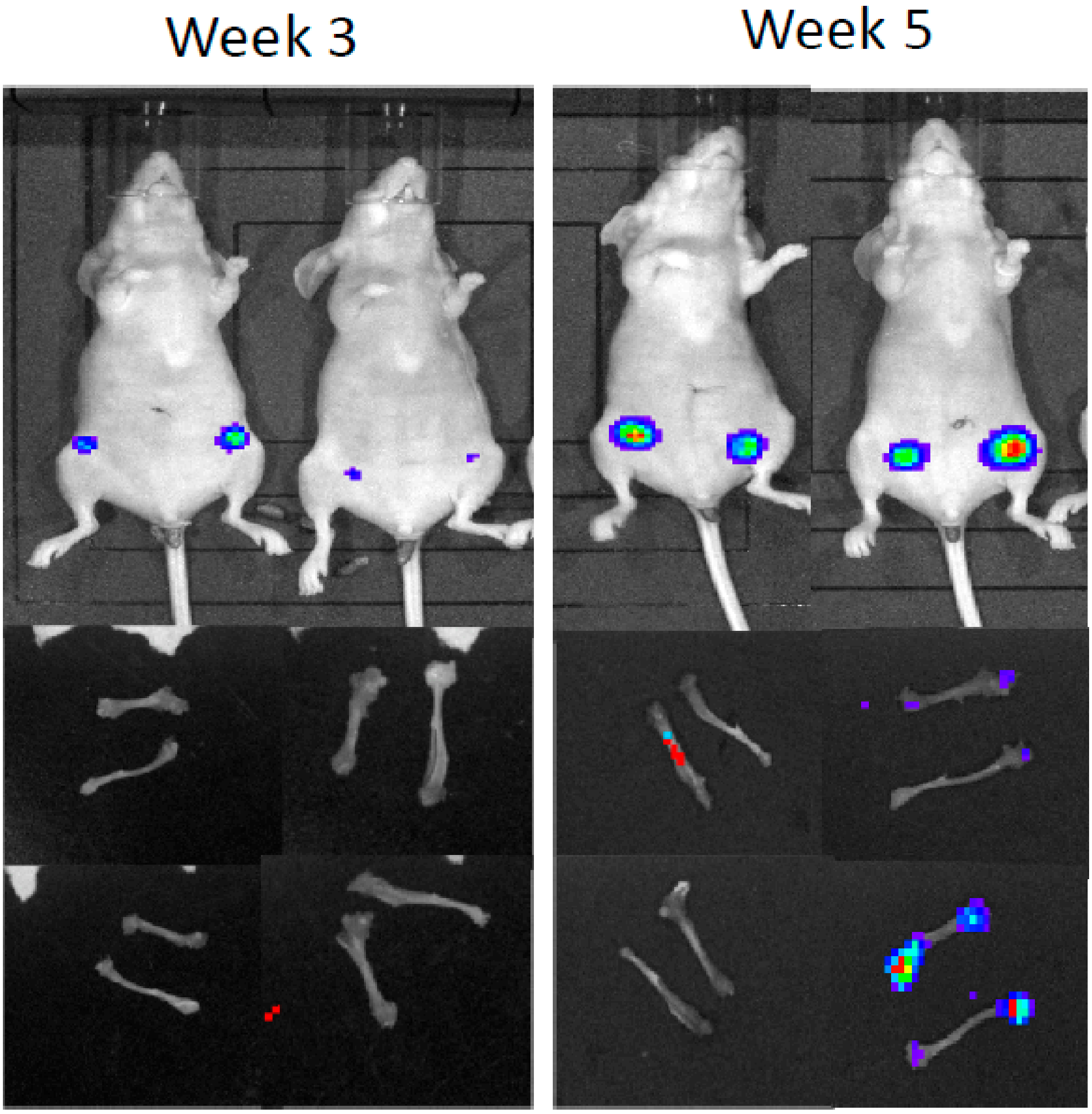

Supplementary Figure S1. Representative images of tumour cell dissemination to the bone 3 and 5 weeks after mammary fat pad injection of T47DLuc2+GFP+ cells.

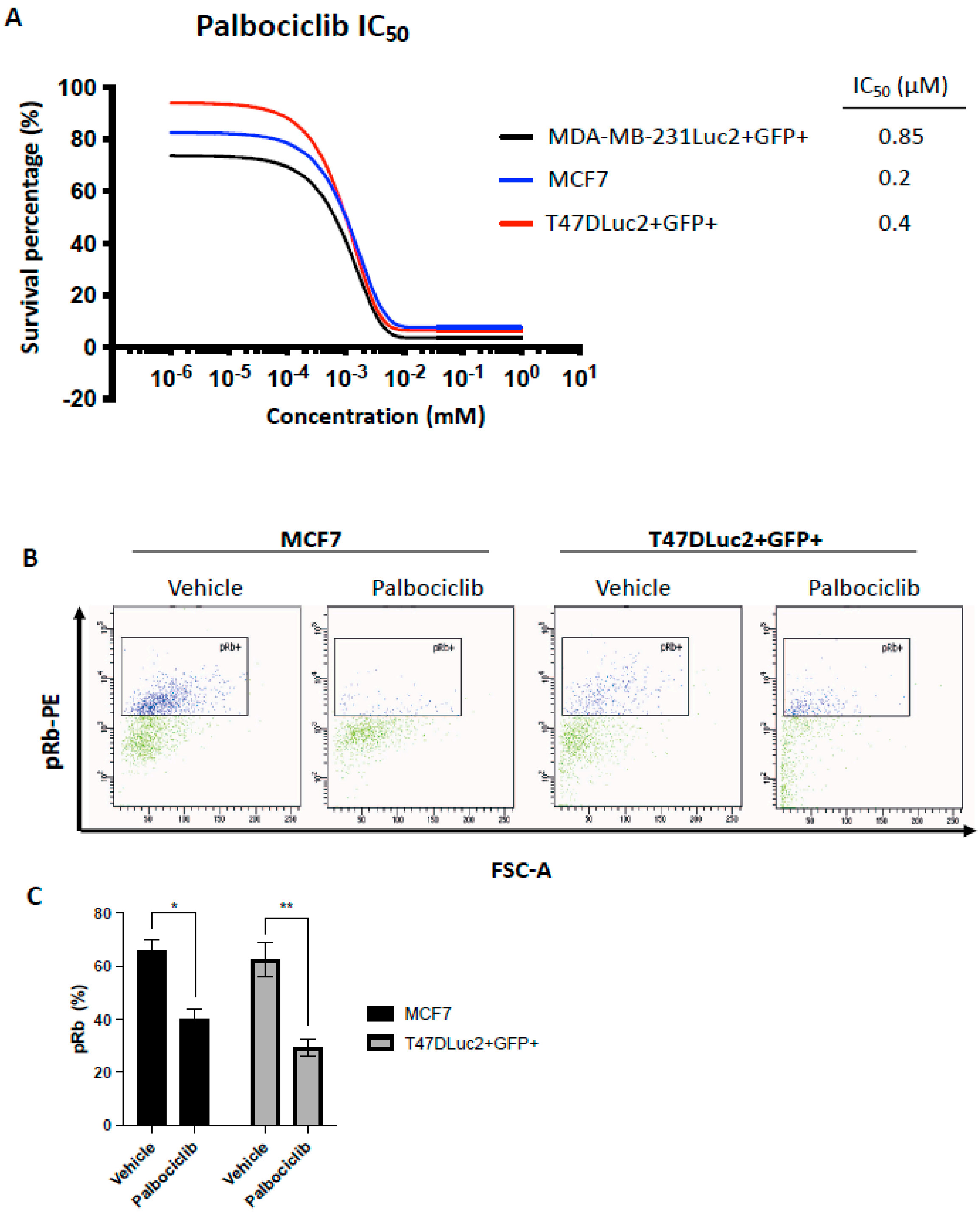

Supplementary Figure S2. (A) Dose response curves in the ER+ve, MCF7 and T47DLuc2+GFP+ cell lines. (B,C) Representative plots of flow cytometry analysis of pRb protein (mean ± SEM, $n = 3$, ** $p < 0.05$; t-test).

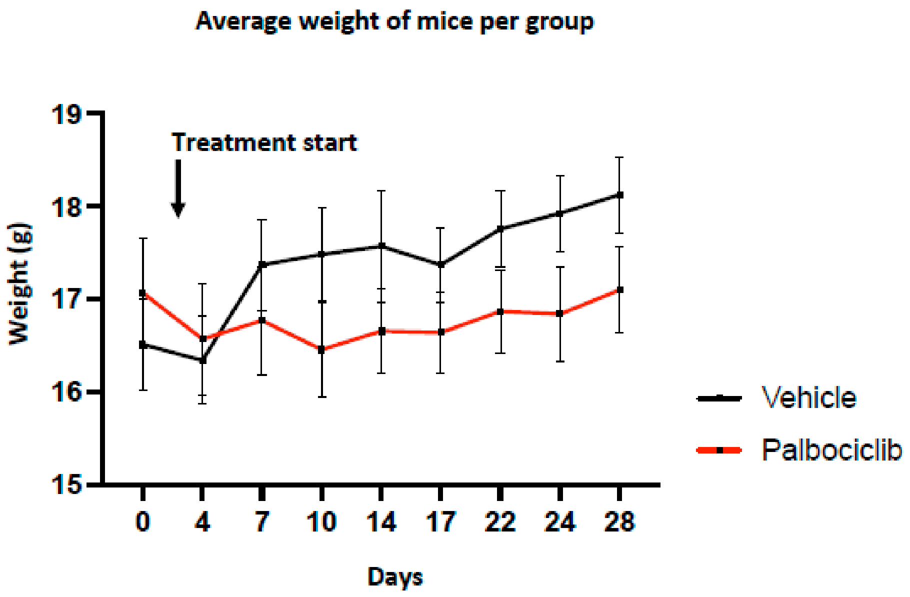

Supplementary Figure S3. Average body weights of mice treated will 100 µl vehicle (Na lactate) or 100 µl palbociclib (100 mg/kg).

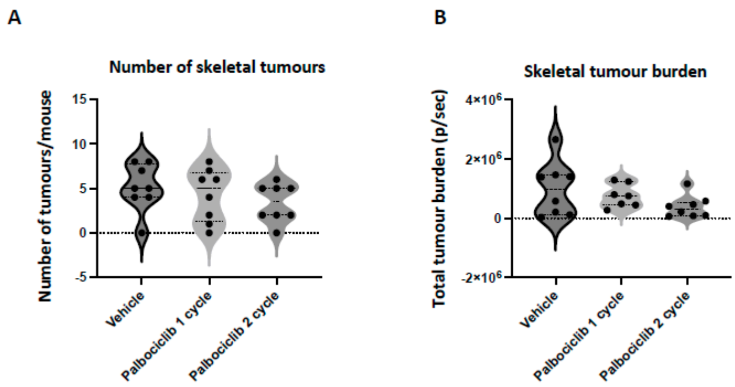

Scheme 4. (A) The number of skeletal tumours and corresponding (B) skeletal tumour burden 2 weeks after intracardiac injection of MDA-MB-231Luc2+GFP+ cells.

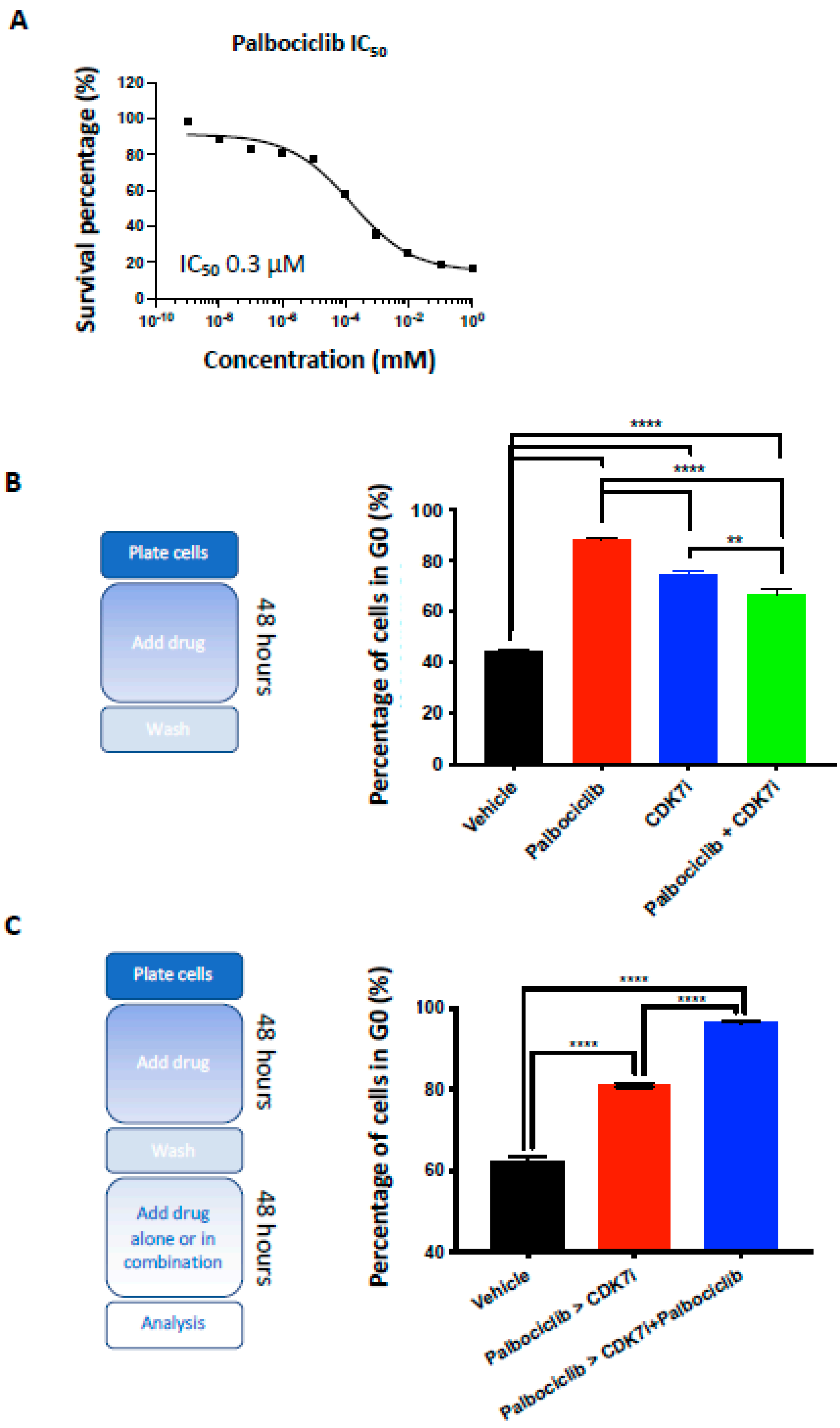

Supplementary Figure S5. (A) The dose response curve and the calculated IC_{50} of CDK7i on the MDA-MB-231Luc2+GFP+ cell line. (B) A total of 4×10^4 MDA-MB-231Luc2+GFP+ cells were treated with drug and incubated for 48 h. Cell cycle analysis was conducted after 48 h exposure time point using PI staining and flow cytometry (mean ± SEM compared to vehicle, $n = 3$, ** $p < 0.005$, **** $p < 0.0005$; t-test). (C) A total of 4×10^4 MDA-MB-231 cells were treated with palbociclib and incubated for 48 h. Cells were then washed and treated with either CDK7i or CDK7i + palbociclib for a further 48 h. At this point, flow cytometry analysis of the cell cycle was conducted by PI staining (mean ± SEM compared to vehicle ,$n = 3$, **** $p < 0.0005$; t-test). Concentrations of palbociclib andCDK7i were 0.4 µM and 0.33 µM, respectively.

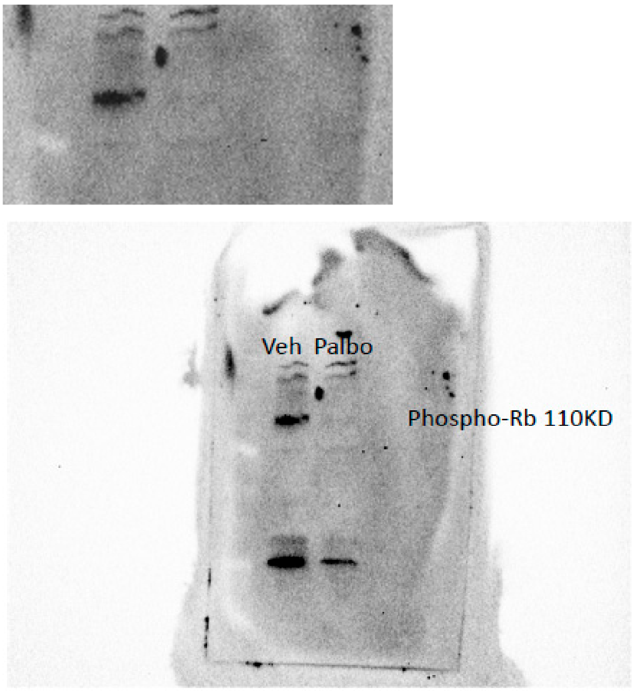

pRb

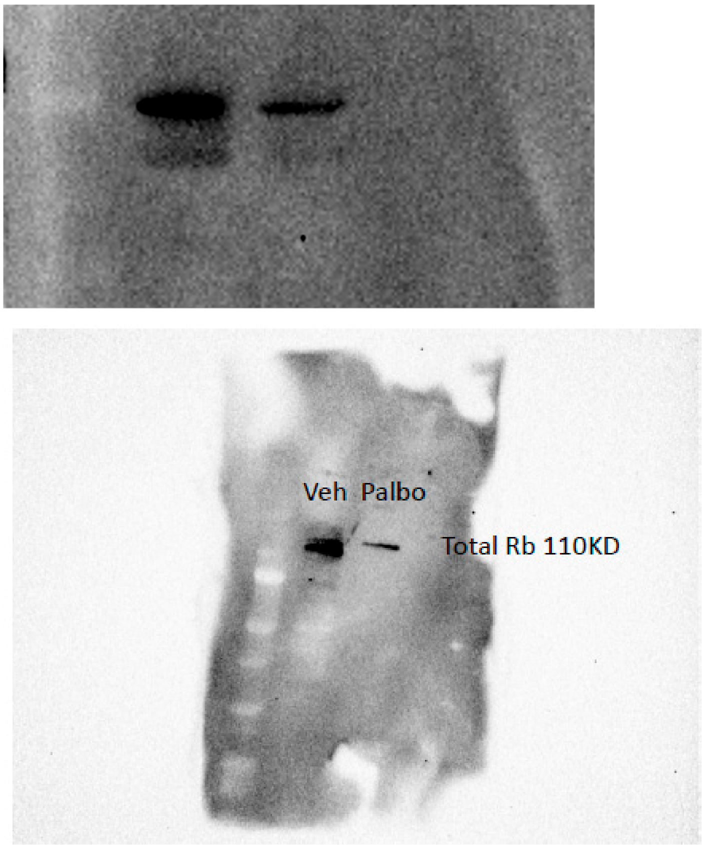

total Rb

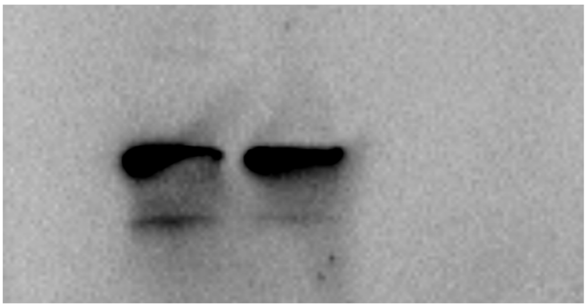

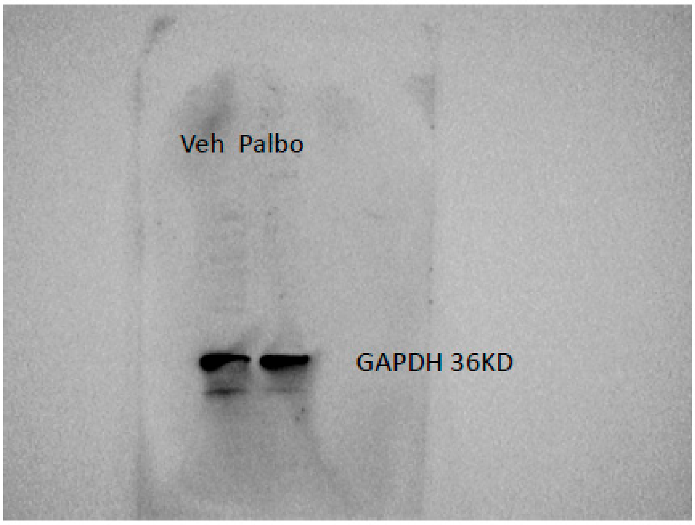

GAPDH

	GAPDH_Merged_UOS 2023-02-23_11h11m03s+UOS 2023-02-22_12h08m07s_Exposure_120.0sec										
	**Lane**	**Band No.**	**Band Label**	**Mol. Wt. (KDa)**	**Relative Front**	**Adj. Volume (Int)**	**Volume (Int)**	**Abs. Quant.**	**Rel. Quant.**	**Band %**	**Lane %**
V_pRb	1	1		N/A	0.317308	11025648	37760832	N/A	N/A	35.87999	22.95828
V_GAPDH	1	2		N/A	0.730769	11932272	33449076	N/A	N/A	38.83035	24.8461
V_Rb	1	3		N/A	0.822115	7771320	25168428	N/A	N/A	25.28966	16.18192
P_pRb	2	1		N/A	0.322115	2829791	17963789	N/A	N/A	14.87344	7.003948
P_GAPDH	2	2		N/A	0.735577	11484319	30745771	N/A	N/A	60.36183	28.42456
P_Rb	2	3		N/A	0.826923	4711688	13654505	N/A	N/A	24.76473	11.66179

Supplementary Figure S6. Original Western blot of Figure 1B.
